# Ten Minutes of α-tACS and Ambient Illumination Independently Modulate EEG α-Power

**DOI:** 10.3389/fnhum.2017.00257

**Published:** 2017-05-18

**Authors:** Heiko I. Stecher, Tania M. Pollok, Daniel Strüber, Fabian Sobotka, Christoph S. Herrmann

**Affiliations:** ^1^Experimental Psychology Lab, Department of Psychology, European Medical School, Cluster for Excellence “Hearing for All”, Carl von Ossietzky UniversityOldenburg, Germany; ^2^Research Center Neurosensory Science, Carl von Ossietzky UniversityOldenburg, Germany; ^3^Division of Epidemiology and Biometry, Department of Health Services Research, Carl von Ossietzky UniversityOldenburg, Germany

**Keywords:** transcranial alternating current stimulation (tACS), EEG, after effect, alpha oscillations

## Abstract

Transcranial alternating current stimulation (tACS) sees increased use in neurosciences as a tool for the exploration of brain oscillations. It has been shown that tACS stimulation in specific frequency bands can result in aftereffects of modulated oscillatory brain activity that persist after the stimulation has ended. The general relationship between persistency of the effect and duration of stimulation is sparsely investigated but previous research has shown that the occurrence of tACS aftereffects depends on the brain state before and during stimulation. Early alpha neurofeedback research suggests that particularly in the alpha band the responsiveness to a manipulation depends on the ambient illumination during measurement. Therefore, in the present study we assessed the brain’s susceptibility to tACS at the individual alpha frequency during darkness compared to ambient illumination. We measured alpha power after 10 min of stimulation in 30 participants while they continuously performed a visual vigilance task. Our results show that immediately after stimulation, the alpha power in the illumination condition for both the stimulated and sham group has increased by only about 7%, compared to about 20% in both groups in the ‘dark’ condition. For the group that did not receive stimulation, the power in darkness remained stable after stimulation, whereas the power in light increased by an additional 10% during the next 30 min. For the group that did receive stimulation, alpha power during these 30 min increased by another 11% in light and 22% in darkness. Since alpha power already increased by about 10% without stimulation, the effect of illumination does not seem to have interacted with the effect of stimulation. Instead, both effects seem to have added up linearly. Although our findings do not show that illumination-induced differences in oscillatory activity influence the susceptibility toward tACS, they stress the importance of controlling for factors like ambient light that might add an independent increase or decrease to the power of brain oscillations during periods, where possible persistent effects of stimulation are explored.

## Introduction

The nature of rhythmic brain activity has been the subject of research since the first use of electroencephalography. While many studies in the past have shown links between specific cognitive tasks and modulations in endogenous frequencies, most were limited to showing purely correlative relationships (Buzsaki et al., 2004). Recent intervention approaches of exploring the role of brain rhythms involve the external modulation of endogenous oscillation by non-invasive brain stimulation like visual flicker (Notbohm et al., 2016), transcranial magnetic stimulation (TMS) (Thut et al., 2012) or transcranial electric stimulation (TES) (Neuling et al., 2013). Among these techniques, transcranial alternating current stimulation (tACS) has proven to be a viable tool that offers direct stimulation of targeted cortical areas in specific frequencies. TACS modulates activity in the cortex by applying sinusoidal currents (or other waveforms) at the scalp (Antal and Paulus, 2013; Herrmann et al., 2013). tACS is thought to cause its effects by interfering with naturally occurring oscillations of brain activity by the mechanism of entrainment [i.e., synchronization of one oscillator to an external one by weak coupling (Pikovsky et al., 2003)]. This has been shown in modeling approaches and animal studies (Fröhlich and McCormick, 2010; Ali et al., 2013) and there is evidence that tACS can modulate frequencies in human EEG (Helfrich et al., 2014b; Cecere et al., 2015).

Many studies have demonstrated that tACS modulates perception (Brignani et al., 2013; Helfrich et al., 2014a; Strüber et al., 2014), short-term memory (Vosskuhl et al., 2015) and motor-excitability (Antal et al., 2008; Bergmann et al., 2009). Multiple studies have shown that tACS also creates persistent physiological effects, like elevated power or coherence of brain oscillations following oscillatory TES (see Veniero et al., 2015 for an elaborate summary). Most tACS studies used the alpha band to demonstrate aftereffects of stimulation. After 10 min of stimulation at occipito-parietal sites at the individual alpha frequency (IAF), Zaehle et al. (2010) reported a frequency specific elevation of alpha power in the EEG. When stimulating for 20 min, this aftereffect has been shown to persist for up to 70 min (Kasten et al., 2016).

The occurrence of stimulation induced effects, however, is not universal. Feurra et al. (2013) could show that excitation of motor evoked potentials was modulated by different tACS frequencies, dependent on mental state (motor imagery or quiescence). Exploration of online-tACS effects in MEG source space showed that phase coherence between resting state alpha and stimulation was increased during states with eyes open only, but not during states with eyes closed (Ruhnau et al., 2016). Another study also found the aftereffect of tACS to be dependent on the brain state. An EEG-experiment with stimulation at IAF produced a robust aftereffect of alpha power increase in participants with open eyes, whereas no such increase was found with eyes closed during the experiment (Neuling et al., 2013). The authors suggested that the alpha activity during closed eyes could be at an un-amplifiable ceiling level, or, alternatively, that the endogenous oscillation was too strong to be influenced by the weak current of tACS (Neuling et al., 2013). Yet another alternative could be that eyes-open and eyes-closed alpha likely involve different physiological mechanisms (Barry et al., 2007) –only one of which was entrained by tACS.

It has been shown that alpha activity with eyes open can be influenced by ambient illumination. For instance, bright illumination reduced alpha activity during a sustained attention task, whereas a dark environment led to an increase in alpha activity (Min et al., 2013). Other early studies on alpha neurofeedback found a strong influence of ambient illumination on the effectiveness of alpha neurofeedback training. Paskewitz and Orne (1973) and Cram et al. (1977) could both show that ambient lighting yielded the biggest effect in alpha increase compared to darkness and bright illumination during a task of operant alpha production.

Taking the state dependency of tACS and the effect of illumination on alpha modulation into account, this study aims to explore how the aftereffect of tACS depends on the illumination-induced state of the endogenous alpha oscillation before, during and after stimulation. To this end, we measured alpha power before and after tACS while the participants executed a visual vigilance task in either a dimly illuminated or a dark room. We hypothesized that we will reproduce the known aftereffect (Zaehle et al., 2010) in a state of weak endogenous alpha during ambient illumination (Min et al., 2013), superimposed on the normal increase of alpha during prolonged states of wakefulness (Cajochen et al., 1995). In contrast, we expect a dark environment to result in stronger endogenous alpha which may not be susceptible to further enhancement via tACS, similar to the state of alpha during eyes-closed (Neuling et al., 2013; Ruhnau et al., 2016).

## Materials and Methods

### Participants

Thirty-three right-handed volunteers (16 females with an average age of 23.8 years, *SD* = 5), participated in the study and gave their written informed consent to participate and have their results anonymously published and received a monetary compensation for their participation. All participants had normal or corrected to normal vision and reported no history of psychiatric or neurological diseases. The study protocol was designed and performed according to the declaration of Helsinki and was approved by the local ethics committee of the Carl von Ossietzky Universität Oldenburg. Two measurements were aborted due to failure to comply with experimental procedure. Data of one participant was omitted from further analyses due to an extreme alpha increase in the post-stimulation period (exceeding 3 σ of the whole sample’s z-scored values). Aborted measurements were repeated with new participants.

### EEG Recording

**FIGURE 1:**
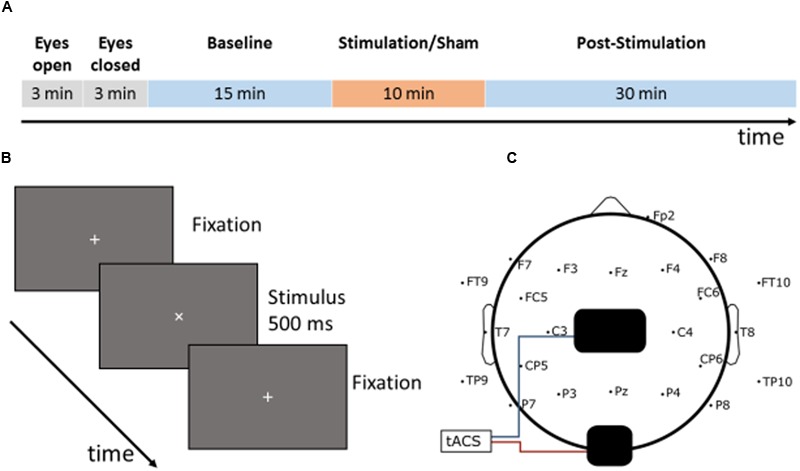
**Experimental setup: (A)** time course of the experiment: a single session started with two 3-min recordings to determine the individual alpha frequency (IAF) once with eyes open and once with eyes closed in a relaxed state. Following this, the participants had to conduct the visual vigilance task for a total duration of 55 min (15 min baseline, 10 min stimulation/sham, 30 min post-stimulation). 15 min after the start of the task, the participants received either 10 min of tACS or sham stimulation at their IAF with an amplitude of 1 mA. **(B)** Visual vigilance: the participants had to fixate a small cross in the middle of the screen during the whole experiment. Every 35–45 s the fixation cross rotated by 45° and stayed rotated for 500 ms. The participants had to detect this rotation and to respond by pressing a button with their right index finger. **(C)** Setup of the tACS electrodes and the EEG electrodes: a 5 cm × 7 cm electrode was placed on Cz and a smaller 4.5 cm × 4.5 cm electrode on Oz according to the international 10/10 system.

The EEG data was measured using a 32 channel actiCHamp amplifier (Brain Products GmbH, Gilching, Germany) with active electrodes in unipolar configuration. The reference electrode was placed at Fp1 and the ground electrode at FPz. Data was recorded using Pycorder (Brain Products GmbH, Gilching, Germany) at an acquisition rate of 10 kHz. Electrodes at 23 head locations according to the 10/10-system were used in the recording, leaving locations beneath the stimulation electrodes empty (see **Figure 1C**). One electrode placed underneath the right eye served as a vertical EOG channel. No online filters were applied. Impedances were brought below 20 kOhm.

### Electrical Stimulation

Transcranial alternating current stimulation was applied using a Neuroconn DC Plus Stimulator (Neuroconn, Ilmenau, Germany) and two rubber electrodes. A 5 cm × 7 cm electrode was placed on Cz, a second 4.5 cm × 4.5 cm electrode on Oz to achieve a maximum of posterior stimulation in accordance with previous experiments (see **Figure 1C**; Neuling et al., 2013; Kasten et al., 2016). The electrodes were affixed to the scalp using Ten20 conductive paste (D.O. Weaver, Aurora, CO, United States) and the impedances were brought below 10 kOhm (mean impedance 3.7 kOhm). Before starting the experiment, it was ensured that each participant was comfortable with a stimulation current of 1 mA peak to peak and did not experience pain, tingling or other unpleasant sensations. For each participant a sinusoidal stimulation at pre-determined IAF was applied. The signal was computed using MATLAB 2016b (The MathWorks, Inc., Natick, MA, United States), and generated using a DAQ-module Ni USB 6229 (National Instruments, Austin, TX, United States) at 10 kHz, then fed into the stimulator via its remote access port. The NiDAQ was externally clocked by the actiCHamp EEG amplifier. The total stimulation duration was 10 min. In accordance to previous studies (Neuling et al., 2013; Kasten et al., 2016), the stimulation started with a linear fade-in of 10 s from 0 to 1 mA amplitude and ended with a linear fade-out of 10 s. The sham stimulation consisted of a 10 s linear fade-in, 10 s of stimulation at 1 mA, followed by a 10 s linear fade-out.

### Procedure

**FIGURE 2:**
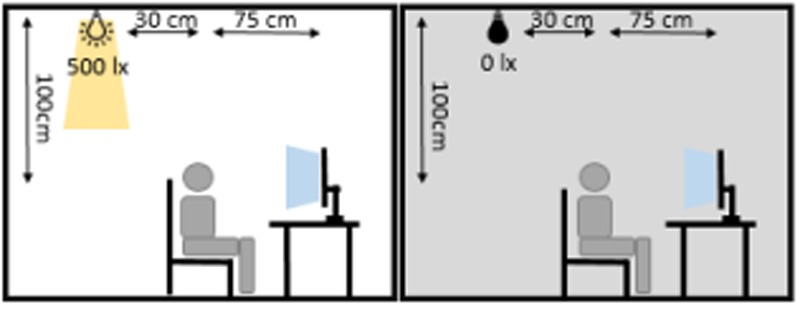
**Illumination conditions during experimental sessions.** The participant was seated 75 cm in front of the monitor. 30 cm behind and 100 cm above the participant’s head, an LED spotlight was positioned. During the ‘dark’-session the spotlight was turned off and the monitor constituted the only light source in the room. During the ‘light’-session, the spotlight was turned on and produced 500 lx at a distance of 1 m.

The experiment consisted of two separate sessions: one with ambient illumination in the lab and one without, in the following denoted ‘light’ and ‘dark.’ Every participant took part in both sessions (50% ‘light’ at first day, 50% ‘dark’ at first day) with an interval of at least 3 days between both sessions to avoid carry-over effects. During the session without ambient illumination, all light sources except the computer monitor for stimulus presentation were turned off. During the session with ambient illumination a 50 W spotlight, positioned in the ceiling thirty centimeters behind the participant, was switched on and dimmed to have an intensity of 500 lx at 1 m distance (height of the participant’s head, see **Figure 2**). Participants were seated in a comfortable chair, 75 cm in front of a Samsung P2470H monitor running at 60 Hz. After preparation of the EEG cap and stimulation electrodes, each session started with a 3-min block resting-EEG with open eyes followed by a 3-min block resting EEG with eyes closed.

The IAF for each participant was determined before the experiment by using the unfiltered 3 min recording during opened eyes, dividing it into 1-s epochs and scanning for the power peak between 8 and 12 Hz at electrode Pz. If the eyes-open recording did not yield a clear alpha peak (this was the case in 20 out of 60 measurements), we used the peak obtained from the eyes-closed recording as the stimulation frequency, since the frequency of the two peaks correlated significantly in the other 40 measurements (*r* = 0.63, *p* < 0.001).

One session of the experiment lasted 55 min during which participants were required to fixate a white 7 mm fixation cross (0.535 vis. deg.), on a gray background (54 cd/m^2^). To keep the participants alert, they had to indicate rotations of the fixation cross (45°, 500 ms duration) occurring every 35–45 s by pressing a button with their right index finger (**Figure 1B**).

Visual stimulation and timing of the experiment were controlled with the Psychophysics toolbox (Brainard, 1997; Pelli, 1997; Kleiner et al., 2007) for MATLAB. EEG was recorded throughout the complete duration of the session. The first 15 min served as a pre-stimulation measurement, followed by a 10-min block of stimulation or sham-stimulation and 30 min of post-stimulation measurement (**Figure 1A**).

Fifty percent of the participants were randomly assigned to receive sham-stimulation. The resulting gender distribution was eight females in the stimulation group and seven in the sham group. The mean age of the resulting stim group was 24.2 years (*SD* : 4.4) and 24.3 years (*SD* : 5.6) in the sham group. Each participant took part in both sessions on different days with two contrasting illumination conditions, with 50% being randomly assigned to start with the second condition on the first day. The second session for each participant always took place at the same time of day as the first session. In both the sham and stimulation group, seven participants were measured in the morning and eight in the afternoon. After each session, participants filled out an adverse effect questionnaire (Brunoni et al., 2011) to indicate whether they experienced any of the common 10 side effects: headache, neck pain, skin irritation, tingling, itching, burning sensation, reddening of the skin, tiredness, trouble concentrating, and mood changes. In addition, it was asked whether they believed to have received stimulation. Participants rated the intensity of each effect on a scale from one to four (1 – none, 2 – mild, 3 – moderate, 4 – severe) and whether they attributed the occurrence to the stimulation (1 – no, 2 – remote, 3 – probable, 4 – definite).

### Data Processing

The EEG data was down-sampled to 500 Hz, high-pass filtered at 1 Hz and low-pass filtered at 100 Hz and re-referenced to a combined Fp1/Fp2 reference using MATLAB and the Fieldtrip toolbox (Oostenveld et al., 2011). Subsequently, the data was cut into two baseline blocks with a length of 5 min (15–10 min and 5–0 min before stimulation) and 30 blocks after stimulation of 1 min each. Eye blinks and eye movement artifacts were removed from the data, using an independent component analysis by manually rejecting the respective components. The blocks were then subdivided into 1-s segments and further DC-jumps and strong muscle artifacts were rejected by identifying all segments that presented a difference between minima and maxima of at least 150 μV. The first 270 artifact-free segments of the baseline blocks and 55 artifact-free segments of each block after stimulation were then used for further analysis. FFT-spectra were calculated for each segment using a Hanning window with 2 s zero-padding. FFT-spectra were then averaged across all segments for each block.

To compensate for a shift in the IAF over the course of the session since the initial determination of the stimulation frequency, the IAF for the post-stimulation power was determined by scanning for the power peak between 8 and 12 Hz in steps of 0.5 Hz at electrode Pz, averaged over the whole 30 min after the stimulation. The mean power of the IAF ± 2 Hz band averaged over all parietal electrodes was then used for further analysis.

According to the laws of synchronization (Pikovsky et al., 2003), even for a mismatch between stimulation frequency and IAF, we would still expect entrainment of the endogenous alpha oscillation, albeit weaker than with a mismatch of zero as has been shown in visual flicker experiments (Schwab et al., 2006). Such a mismatch can also influence the aftereffect of tACS (Vossen et al., 2015). To include the effects of small mismatches on the aftereffect, we added the factor mismatch to our analysis. As the relationship between strength of entrainment and frequency is non-linear (Notbohm et al., 2016) with the strongest entrainment centered on the eigenfrequency of the driven oscillator, we used only the absolute value of the mismatch.

### Statistical Analysis

Statistical analysis was performed using SPSS 24.0 (IBMCorp, Armonk, NY, United States) and R 3.3.0 (R Foundation for Statistical Computing, Vienna, Austria) employing the mgcv-package (Wood, 2006). Behavioral data analysis was conducted on accuracy (i.e., percent correct responses) and reaction times with a repeated measures ANOVA on the within-subjects factors *time* (baseline, stimulation, 0–15 min after stimulation, 16–30 min after stimulation) *illumination* (‘light’ vs. ‘dark’) and the between-subjects factor *group* (stimulation vs. sham). Greenhouse–Geisser correction was applied where appropriate. Differences in adverse effect between stimulation and sham group were tested using a Wilcoxon-Mann-Whitney-*U* test. Differences in answering the question of believing to have received stimulation were assessed using a Chi-squared test.

Changes in alpha power before stimulation were explored by comparing the absolute power values of the average 15–10 min (baseline 1) before stimulation onset with the average of 5–0 min (baseline 2) before stimulation onset in a repeated measures ANOVA with the within-subjects factor *time* (baseline 1 vs. baseline 2) and *illumination* (‘light’ vs. ‘dark’), pooled over stimulation and sham group.

For the analysis of the aftereffect, all power values were normalized to the second baseline (5–0 min before stimulation). We explored the development of alpha power over time in the post-stimulation period by using a generalized additive mixed regression model (GAMM) in order to account for inter-subject variability and for time being a continuous, multi-level variable. The time period after the end of stimulation for which the aftereffect was analyzed lasted 30 min. If alpha values from 1800 spectra (30 min times 60 s) were entered into an ANOVA as a factor time with 1800 levels, this would most likely not yield significant results due to the huge number of degrees of freedom. Previous studies have circumvented this problem by averaging over adjacent time seconds in order reduce the number of levels in the ANOVA (Neuling et al., 2013; Kasten et al., 2016). A GAMM, however, adequately takes the multi-level factor time into account. As the distribution of the alpha values was strictly positive and right-skewed, a Gamma likelihood with an identity link was used in the model. The factors time, illumination, stimulation, frequency-mismatch, and day of measurement (1st or 2nd) were included as covariates and all pairwise interaction terms were constructed in order to gain a saturated model as a starting point. Three-way interactions were not considered because their interpretation is problematic. From this saturated initial model, we performed a manual model selection based on the Akaike Information Criterion (AIC) to obtain the optimal regression predictor with respect to model fit and complexity (See Supplementary Table for a selection of tested models). Further, the model included a random effect for a participant’s ID, a random effect of time and a random effect of illumination. As multiple data points were collected subsequently for each individual, we needed to assume a dependency between measurements of the same participant. For the random effects, we applied an auto-correlated covariance structure of order 1 per ID and illumination scenario.

## Results

### Behavioral Results

**FIGURE 3:**
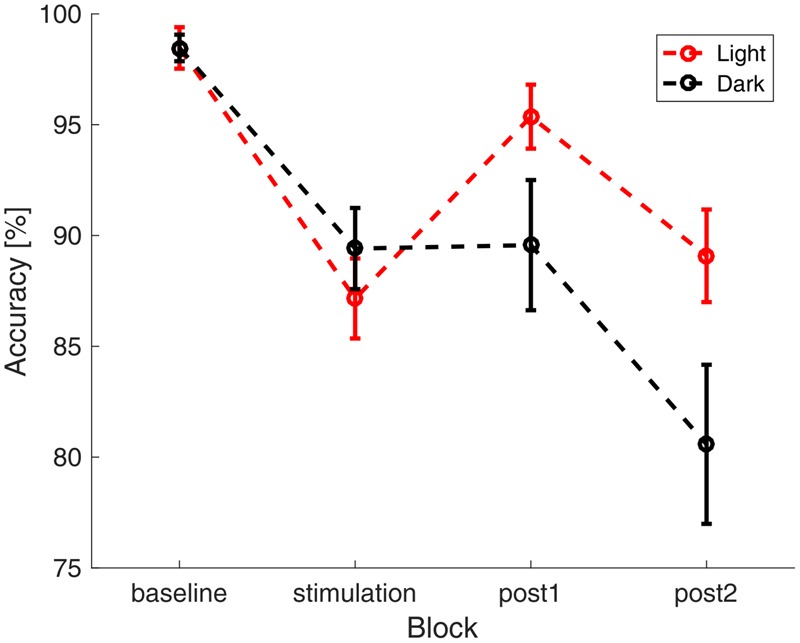
**Accuracy in the visual vigilance task over time.** Average accuracy in the four different blocks of the experiment (baseline, stimulation, 0–15 min after stimulation, 16–30 min after stimulation). The data was pooled over both stimulation and sham group. In red the results during the ‘light’-condition are depicted, black depicts the ‘dark’-condition. The error bars show the standard error of the mean.

The participants reached an average accuracy of 91.2% (*SD*: 8.8%) in the vigilance task with an average reaction time of 503 ms (*SD* : 125 ms), indicating high vigilance of the participants throughout the study. A repeated measures ANOVA with the between factor group and the within factors illumination and time (baseline, stimulation, 0–15 min after stimulation, 16–30 min after stimulation) did not show significant differences in reaction times between groups (*group*: *F*_1,28_ = 0.828, *p* = 0.371, η^2^ = 0.029; *illumination*: *F*_1,28_ = 0.042, *p* = 0.840, η^2^ = 0.001; *time*: *F*_3,26_ = 2.202, *p* = 0.094, η^2^ = 0.073; *group × time: F*_3,26_ = 0.520, *p* = 0.587, η^2^ = 0.018; *illumination × time: F*_3,26_ = 2.318, *p* = 0.81, η^2^ = 0.076; *group × illumination*: *F*_1,28_ = 1.987, *p* = 0.170, η^2^ = 0.066; *group × illumination × time: F*_3,26_ = 0.579, *p* = 0.630, η^2^ = 0.020). A repeated measures ANOVA with the same factors on accuracy revealed a significant effect of time and a significant interaction between ambience and time (*group*: *F*_1,28_ = 0.036, *p* = 0.85, η^2^ = 0.001; *illumination*: *F*_1,28_ = 2.217, *p* = 0.148, η^2^ = 0.073; *time*: *F*_3,26_ = 23.161, *p* < 0.000, η^2^ = 0.453; *group × time: F*_3,26_ = 0.170, *p* = 0.855, η^2^ = 0.006; *illumination × time: F*_3,26_ = 4.448, *p* = 0.013, η^2^ = 0.137; *group × illumination*: *F*_1,28_ = 0, *p* = 0.990, η^2^ = 0; *group × illumination × time: F*_3,26_ = 0.303, *p* = 0.823, η^2^ = 0.011). In order to resolve the *illumination × time* interaction, we performed two-sided *t*-tests comparing the accuracy between illumination conditions in each block. Uncorrected results showed a difference at the last block of time (*baseline: t*_58_ < 0.01, *p* = 1.000, *d* < 0.01; *stimulation: t*_58_ = -0.876, *p* = 0.385, *d* = 0.230; *post1 : t*_58_ = 1.77, *p* = 0.081, *d* = 0.465; *post2 : t*_58_ = 2.04, *p* = 0.045, *d* = 0.537). After applying FDR-correction, this effect did not survive (*baseline: p* = 1.000; *stimulation: p* = 0.513; *post1 : p* = 0.164; *post2 : p* = 0.164). The time course of accuracy, as depicted in **Figure 3** suggests a general decrease of accuracy over time. In order assess the effect of fatigue, we tested the mean single-subject correlations of accuracy and block number against zero. This revealed that accuracy declined with time passed throughout the experiment [*t*(29) = -5.8917, *p* < 0.01].

The answers to the questions whether participants believed to have received stimulation did not differ significantly between groups (stim: 76.67%, sham: 66.67%, χ^2^_1_ = 0.739, *p* = 0.39). The response to the items on the adverse effect questionnaire did not show a significant difference between stimulation and sham group (Mann-Whitney-*U* test: all *p* > 0.05). This indicates that the blinding was successful. Most frequently reported symptoms were tiredness (85%), trouble concentrating (76.67%) and tingling (40%). Only tingling was on average attributed to the stimulation (mean score: 2.5).

### Pre-stimulation Alpha-Increase

**FIGURE 4:**
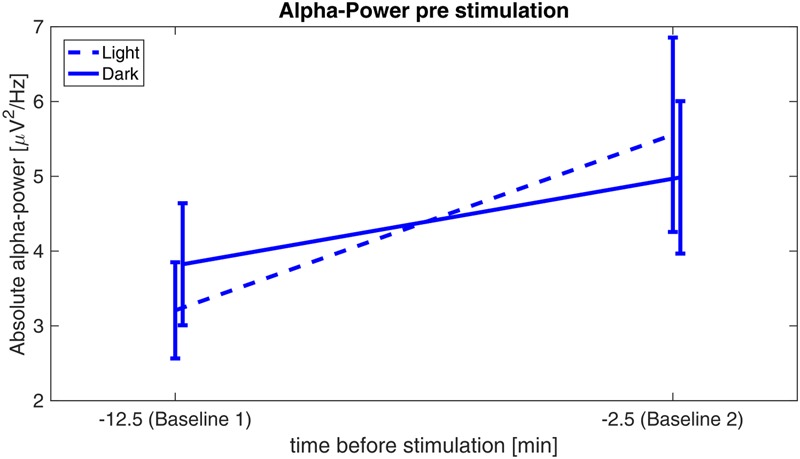
**Alpha-power before stimulation.** Absolute alpha power before stimulation: power values are averaged over the first 5 min (baseline 1) of the experiment and 5 min before onset of the stimulation (baseline 2) for the sessions in ‘light’ (dashed) and in ‘dark’ (solid).

Analyses revealed a significant main effect of *time* (*F*_1,29_ = 12.202, *p* = 0.002, η^2^ = 0.296), whereas the factor *illumination* (*F*_1,29_ = 0.002, *p* = 0.961, η^2^ < 0.001) and the interaction *time × illumination* (*F*_1,29_ = 3.13, *p* = 0.088, η^2^ = 0.097) did not reach significance, indicating a similar increase of pre-stimulation alpha power from baseline 1 to baseline 2 for both illumination conditions (**Figure 4**).

### Aftereffect

**FIGURE 5:**
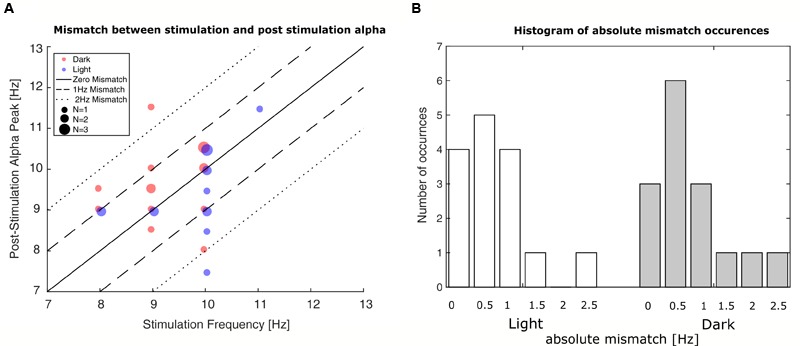
**Occurrences of mismatches between stimulation frequency and post-stimulation alpha frequency. (A)** Plot of stimulation frequency vs. post-stimulation IAF. The smallest dots represent single participants, whereas the bigger dots represent two or three participants. Blue dots represent participants in the ‘light’ condition; red dots represent participants in the ‘dark’ condition. The solid line depicts zero mismatch between stimulation frequency and post-stimulation IAF; the dashed lines depict the areas of ±1 Hz and ±2 Hz deviation. **(B)** Histograms depict number of occurred frequency mismatches between stimulation frequency and post-stimulation IAF. Left bars: ‘light’ condition; right bars: ‘dark’ condition. The mismatch is shown in absolute deviations in Hz in accordance with their inclusion in the GAMM-based analysis.

Evaluation of the mismatch between stimulation-frequency and the IAF of the measurement’s last minute revealed that the initial estimation deviated on average 0.7 Hz in the ‘light’-group and 0.8 Hz in the ‘dark’-group with rare mismatches up to ±2.5 Hz as can be seen in **Figure 5**. To control for effects of the mismatch, it was added as a factor to the GAM-model.

The final model contained the fixed effects factor *illumination* and the interactions *illumination* ^∗^ *frequency*-*mismatch, illumination* ^∗^ *time* and *stimulation* ^∗^ *time* as well as the random effects factors *time* and *illumination.* All other factors and interactions have been removed in order to gain a model of minimal AIC.

The final model predicts relative alpha power post-stimulation according to the following equation:

$$\alpha \;=\beta_{0}\;+\beta_{1\;}\;\;illum+\beta_{2}\;illum\;*\;mmatch+\beta_{3\;\;}\;illum\;*\;time+\beta_{4}$$

$$stim\;*\;time+\gamma_{0,ID}\;+\gamma_{1,ID}\;*\;time\;+\gamma_{2,ID}\;*\;illum+\varepsilon$$

All β-coefficients represent fixed effects, whereas γ-coefficients represent random effects. The coefficients β_0_, β_1_, β_2_, γ_0_, γ_2_ describe the intercept (i.e., the power of the alpha activity immediately after the end of the stimulation period) of the post-stimulation alpha time course depending on the conditions of *illumination, stimulation*, and the *mismatch* between stimulation frequency and IAF as well as random individual effects. The coefficients β_3_, β_4_, and γ_1_ describe the slope (i.e., increase in alpha power over time), depending on *stimulation, illumination* and the random individual effects, while 𝜀 describes the residual error.

**FIGURE 6:**
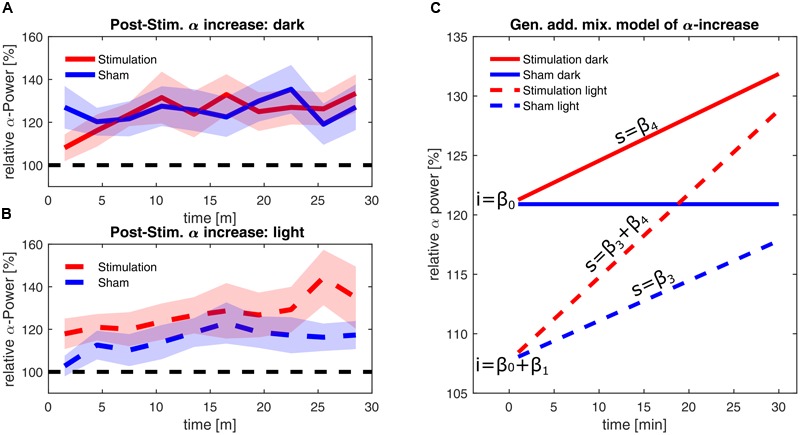
**Alpha-power changes post-stimulation. (A)** Time course of the relative alpha power during the 30 min after stimulation in the ‘dark’ condition. **(B)** Time course of the relative alpha power during the 30 min after stimulation in the ‘light’ condition. Alpha-power is relative to baseline 2, averaged over three min. Stimulation group is shown in red and sham group is shown in blue. The dashed black line represents baseline power. The shaded areas depict standard error of the mean. **(C)** Time course of alpha power as fitted with a GAMM with the fixed effects of time, stimulation and illumination, omitting random effects and the non-significant effect of frequency mismatch. The blue lines depict the sham groups; the red lines depict the stimulation groups. Solid lines depict power in darkness, while dashed lines depict the power in the ‘light’ condition. The letters ‘i’ indicate the intercepts of the time course of alpha, resulting from the coefficient β_0_ in darkness, and the coefficients β_0_+β_1_ in light. The letters ‘s’ indicate the slopes of the time course of alpha, resulting from the coefficients β_3_ and β_4_ (power change over time) in light and due to stimulation, respectively. For stimulation in ambient light, coefficients β_3_ and β_4_ add up, leading to the steepest increase of alpha over time.

The estimators of the final model are listed in **Table 1**, demonstrating a significant effect of *illumination* on alpha power and significant interactions of *illumination × time* and *stimulation × time*. The final model has a marginal *R*^2^ of 0.078, measuring the determination of the fixed effects and a conditional *R*^2^ of 0.999, measuring the determination of both the fixed and the individual random effects. In **Figures 6A,B**, the smoothed time course of the measured power change for a ‘dark’ and ‘light’ ambience are depicted, whereas **Figure 6C** shows the resulting predictions of the model for linear alpha increase in the different groups, omitting the effect of frequency-mismatch which did not reach significance.

**Table 1:** Result summary of final generalized additive mixed model.

Parameter	Coefficient β	*SE* (β)	*t*	*p*
(β_0_) Intercept	120.900	4.782	25.285	<0.001
(β_1_) Illumination	-13.183	6.284	-2.098	0.036
(β_2_) Illumination *×* Freq.- Mismatch	10.965	7.583	1.446	0.148
(β_3_) Illumination *×* Time	0.337	0.155	2.180	0.029
(β_4_) Stimulation *×* Time	0.366	0.171	2.133	0.033

*Coefficient estimates β for the fixed effects, standard Error SE(β), *t*-value *t* and significance level *p*. The model’s has marginal *R*^*2*^ of 0.078 and a conditional *R*^*2*^ of 0.999.*

Immediately after the end of stimulation, both stimulation and sham group in the ‘dark’ condition showed a higher increase in alpha power by 20% compared to baseline (general intercept β_0_). In contrast to this, brighter illumination only shows a smaller increase in alpha power of about 7% within both the stimulation and the sham group (β_0_ + intercept due to illumination β_1_). Within the 30 min post-stimulation period, the alpha power in the sham group remained stable during darkness, whereas alpha power in the ‘light’ condition increased by an additional 0.337% per minute (slope caused by illumination β_3_). In the stimulated groups, the stimulation leads to a general increase of power over time by 0.366% per minute in darkness (slope caused by stimulation β4). Within the stimulated group in ‘light’ this adds up to the illumination-based increase to an increase of 0.7% per minute (slope β3+ β4), resulting in ultimately higher alpha power within the stimulated group, compared to the respective sham group (See Supplementary Figure for a plot containing all individual trajectories).

**FIGURE 7:**
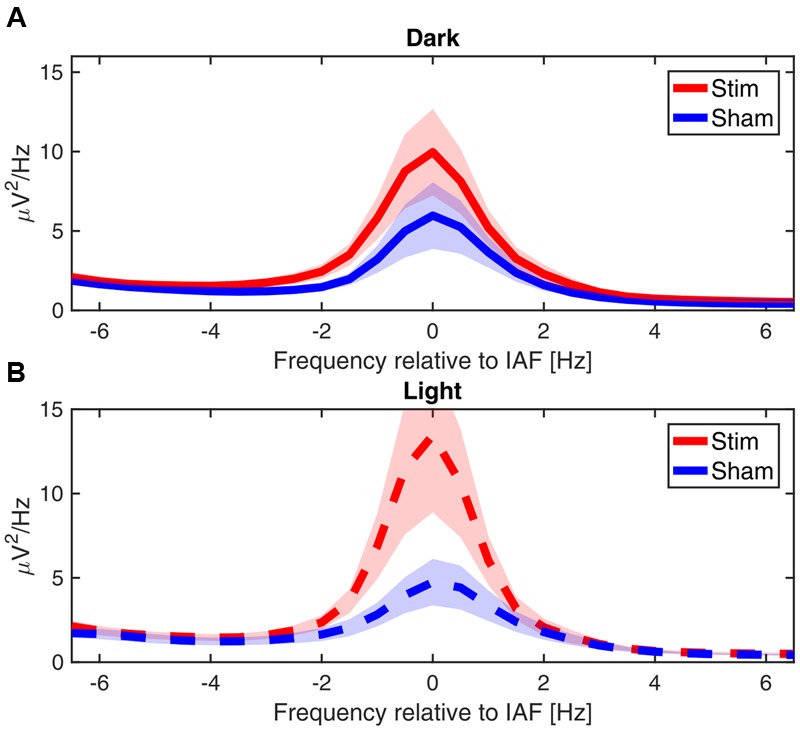
**Power spectra post-stimulation.** Grand average power spectra of the mean alpha activity post-stimulation, centered on the IAF for each participant (peak power between 7.5 and 12 Hz). **(A)** In ‘dark’ **(B)** in ‘light.’ Shaded area shows the standard error of the mean.

While we did not test whether our effects are frequency specific, grand average of the power spectra averaged over the total 30 min of post-stimulation show that the differences between conditions are closely confined to the immediate vicinity of the alpha peak, as can be seen in **Figure 7**.

## Discussion

In this study, we assessed whether 10 min of stimulation at IAF produces an aftereffect of elevated alpha power as has been reported previously for longer stimulation durations. Moreover, we explored the impact of ambient illumination on the occurrence of this aftereffect. Our results show that 10 min of tACS led to an aftereffect in alpha power similar to earlier findings of Zaehle et al. (2010), who found an aftereffect of increased alpha power within the first three min after stimulation. Extending the findings of Zaehle et al. (2010), our results demonstrate a linear increase of alpha power within thirty min after stimulation. Furthermore, our results show that the alpha power immediately after the end of the stimulation period (tACS/sham) depends on ambient illumination level.

We found that the expected decrease of alpha activity in a bright environment (Min et al., 2013) was not present within the first 15 min of our recordings (baseline 1 to baseline 2). Instead, illumination seems to take effect during the stimulation period (tACS/sham), resulting in reduced alpha power at the beginning of the post-stimulation period, i.e., the time course of alpha power after stimulation starts at different levels for the ‘dark’ and the ‘light’ condition. Our results suggest that in the absence of stimulation, there is a general increase in alpha activity in ambient light which is absent in a dark environment. A general increase in alpha activity over time was to be expected, as the continuous task causes increasing mental fatigue, which is a well-known effect (Daniel, 1967; Cajochen et al., 1995; Boksem et al., 2005; Oken et al., 2006).

We could not find evidence that tACS-aftereffect is dependent on illumination. It rather seems that tACS raises the total power level toward which the alpha activity converges, adding linearly to the illumination effect. It seems, that the endogenous alpha in our ‘dark’ condition did not reach a ceiling level above which a further elevation by tACS is no longer possible (Neuling et al., 2013), refuting our initial hypothesis. The ongoing vigilance task probably prevents the fatigue induced alpha activity from reaching a ceiling level that cannot be further increased.

It has been shown, that perception is linked to the activity in the alpha band (Ergenoglu et al., 2004; Hanslmayr et al., 2005; Thut et al., 2006). However, we only found a general decline in the participants’ accuracy over time in the vigilance task, which seemed to be independent of ambience illumination and stimulation. This is in line with an earlier study (Kasten et al., 2016), that used the same visual vigilance task. This effect is probably due to our stimuli being super-threshold and lasting several alpha-cycles, as they were merely designed to keep the participants in a state of sustained attention.

Our findings of a delayed increase in post-stimulation alpha power is in line with studies utilizing 20 min of tACS and a prolonged measurement of post-stimulation activity (Neuling et al., 2013; Kasten et al., 2016). Whereas numerous studies have suggested entrainment as a candidate mechanism during tACS (Neuling et al., 2012, 2015; Helfrich et al., 2014b; Strüber et al., 2014; Witkowski et al., 2016), recent findings of Vossen et al. (2015) could show that the aftereffect is not a manifestation of entrainment echoes. Instead, their findings point toward spike-timing dependent plasticity (STDP, see Feldman, 2012) as the main factor for bringing up tACS aftereffects as was previously suggested by Zaehle et al. (2010). According to Veniero et al. (2015), STDP acts during the entrained state of tACS by causing synapses in recurrent neuronal networks of specific intrinsic frequencies to strengthen their connections by long term potentiation (LTP), whereas others are weakened by long term depression (LTD). Thus, it seems plausible to assume a two-stage process to be responsible for tACS aftereffects to occur: at first, entrainment is responsible for amplitude enhancements of brain oscillations *during* tACS. Second, if entrainment lasts sufficiently long, synaptic plasticity is induced resulting in prolonged amplitude enhancements *after* the end of stimulation. From this point of view, our findings suggest that the ambient illumination influences the natural increase or decrease of alpha activity, whereas the maximum capacity of the underlying networks for alpha activity can be strengthened by tACS.

It is currently unclear how long these changes persist. The natural increase in power during long-lasting experiments (>1 h), ultimately leads to the power of the unstimulated conditions catching up to the level of the stimulated condition, which masks the “real” stimulation effect, as reported by Kasten et al. (2016) for an aftereffect-duration of 70 min. This, however, does not necessarily mean that physiological changes induced by tACS have ceased at this point in time. A major problem in studying tACS aftereffects is the increase of alpha activity caused by fatigue. In order to better control this source of alpha increment, future studies might employ events that naturally diminish the alpha-activity in the post-stimulation period – like a marked change in illumination. This procedure could reveal if a stimulation-induced faster increase in alpha activity is still present and, thereby, help to disentangle fatigue-driven from tACS-induced alpha enhancements.

As the difference in brightness of the two illumination levels that we employed was relatively small, we cannot generalize tACS effect to more drastic differences in illumination like daylight vs. complete darkness. However, given that even small differences in illumination led to significant effects on the natural progression of alpha activity during a sustained task, we strongly suggest to take ambient illumination into consideration when designing alpha modulation studies.

Depending on the overall duration of the experiment and the length of the post-stimulation observation period, very low levels of illumination may raise the alpha activity to high levels, where aftereffects of tACS are no longer visible.

## Author Contributions

HS, TP, DS, CH: designed the study; HS, TP: acquired the data; HS, FS, TP: analyzed the data; HS, TP, FS, DS, CH: wrote the article.

## Conflict of Interest Statement

CH has filed a patent application on brain stimulation and received honoraria as editor from Elsevier Publishers, Amsterdam. The other authors declare that the research was conducted in the absence of any commercial or financial relationships that could be construed as a potential conflict of interest.
