# Supplementary material for: Ten Minutes of α-tACS and Ambient Illumination Independently Modulate EEG α-Power
Source: Front Hum Neurosci. 2017 May 18;11:257. doi: 10.3389/fnhum.2017.00257 (PMC5435819; doi:10.3389/fnhum.2017.00257)
Supplement: Supplementary file 1 [file Data_Sheet_1.PDF]

Supplementary Material

Ten minutes of α-tACS and ambient illumination independently modulate EEG α-power

Heiko I. Stecher[1], Tania M. Pollok[1], Daniel Strüber[1,2], Fabian Sobotka[3], Christoph S. Herrmann[1,2*]

[1] Experimental Psychology Lab, Department of Psychology, European Medical School, Cluster for excellence "Hearing for all", Carl von Ossietzky University, Germany

[2] Research Center Neurosensory Science, Carl von Ossietzky University, Germany

[3] Division of Epidemiology and Biometry, Department of Health Services Research, Carl von Ossietzky University, Germany

*** Correspondence:**
Prof. Dr. Christoph S. Herrmann

Experimental Psychology Lab,

Carl-von-Ossietzky-Universität Oldenburg,

Ammerländer Heerstraße 114-118,

2611 Oldenburg, Germany

Tel.: +49 441 798 4936

Fax: +49 441 798 3865

Email: christoph.herrmann@uni-oldenburg.de

1 Supplementary Figures and Tables

1.1 Supplementary Figures

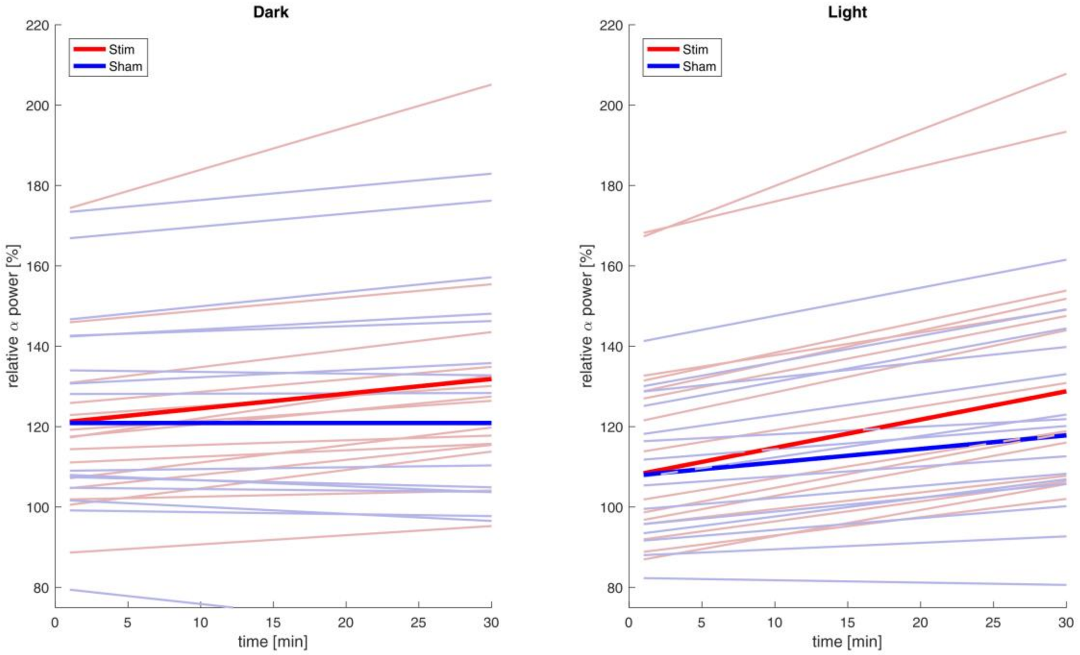

Supplementary Figure: Time course of alpha power as fitted with a GAMM for the fixed effects (bold lines) and time course for each individual with the individual random effects (thin lines). Red are the stimulation groups; blue are the sham groups.

1.2 Supplementary Tables

Supplementary Table

The table shows a selection of different generalized additive mixed models that were tested during the manual model selection process. In the Model column, the formulas with different selections of all factors and interactions are shown. The Random column shows the respective selection of random effects, while the Correlation column depicts the employed autocorrelation. For each tested model the Akaike information criterion is shown, which represents a relative measure for the quality of the model for the given dataset, where lower values represent less information loss. The final model, that was selected for our statistics can be found at the bottom.

Model	Random	Correlation	AIC
Mismatch:Illu + Illu + Illu:Time + Time:Stim	ID = ~ Time,Illu = ~ 1	-	177260.19
Mismatch:Illu + Illu + Illu:Time + Time:Stim	ID =~ 1	corAR1(form=~Time|ID/Illu)	17892.16
Mismatch + Illu:Time:Stim	ID = ~ Time,Illu = ~ 1	corAR1(form=~Time|ID/Illu)	17713.19
Mismatch:Illu + Illu + Illu:Time + Time + Stim + Time:Stim	ID = ~ Time,Illu = ~ 1	corAR1(form=~Time|ID/Illu)	17709.53
Illu + Illu:Time + Time:Stim	ID = ~ Time,Illu = ~ 1	corAR1(form=~Time|ID/Illu)	17707.90
Mismatch:Illu + Illu + Illu:Time + Time:Stim	ID = ~ Time,Illu = ~ 1	corAR1(form=~Time|ID/Illu)	17707.83
